# RNA-Seq derived identification of differential transcription in the chrysanthemum leaf following inoculation with *Alternaria tenuissima*

**DOI:** 10.1186/1471-2164-15-9

**Published:** 2014-01-04

**Authors:** Huiyun Li, Sumei Chen, Aiping Song, Haibin Wang, Weimin Fang, Zhiyong Guan, Jiafu Jiang, Fadi Chen

**Affiliations:** 1 College of Horticulture, Nanjing Agricultural University, Nanjing 210095, China; 2 Jiangsu Province Engineering Lab for Modern Facility Agriculture Technology and Equipment, Nanjing 210095, China

**Keywords:** Ornamental plant, *Alternaria tenuissima*, RNA-Seq, Cell wall modification genes

## Abstract

**Background:**

A major production constraint on the important ornamental species chrysanthemum is black spot which is caused by the necrotrophic fungus *Alternaria tenuissima.* The molecular basis of host resistance to *A. tenuissima* has not been studied as yet in any detail. Here, high throughput sequencing was taken to characterize the transcriptomic response of the chrysanthemum leaf to *A. tenuissima* inoculation.

**Results:**

The transcriptomic data was acquired using RNA-Seq technology, based on the Illumina HiSeq™ 2000 platform. Four different libraries derived from two sets of leaves harvested from either inoculated or mock-inoculated plants were characterized. Over seven million clean reads were generated from each library, each corresponding to a coverage of >350,000 nt. About 70% of the reads could be mapped to a set of chrysanthemum unigenes. Read frequency was used as a measure of transcript abundance and therefore as an identifier of differential transcription in the four libraries. The differentially transcribed genes identified were involved in photosynthesis, pathogen recognition, reactive oxygen species generation, cell wall modification and phytohormone signalling; in addition, a number of varied transcription factors were identified. A selection of 23 of the genes was transcription-profiled using quantitative RT-PCR to validate the RNA-Seq output.

**Conclusions:**

A substantial body of chrysanthemum transcriptomic sequence was generated, which led to a number of insights into the molecular basis of the host response to *A. tenuissima* infection. Although most of the differentially transcribed genes were up-regulated by the presence of the pathogen, those involved in photosynthesis were down-regulated.

## Background

Chrysanthemum (*Chrysanthemum morifolium* Ramat.) is the second most commercially valuable ornamental species after rose [1,2]. A serious production constraint is represented by black spot disease (causative pathogen the necrotrophic fungus *Alternaria tenuissima* (Fr.) Wiltsh) [3]. The disease is most damaging during humid, warm conditions, which makes it a year-round problem for greenhouse-based production [3,4]. Severe infections damage the commercial value of the plant, as they cause leaf necrosis, and reduce the quantity and quality of the flowers [5]. Little is known regarding the chrysanthemum/black spot host-pathogen interaction. However, in the host-pathogen system involving the model species *Arabidopsis thaliana* and the related pathogen *A. brassicicola,* the pathogenesis-related protein PR4 is significantly up-regulated [6]. In the tomato/*A. alternata* system, the ethylene (ET), jasmonate (JA) and salicylic acid (SA) signalling pathways are all activated as part of the host response [7], while the response of mint to *A. alternata* infection features many proteins related to stress and defence [8]. Finally, Egusa *et al.* (2009) [9] have shown that the transcription of the genes *PGIP* (polygalacturonase inhibiting protein) and *PPO* (polyphenol oxidase) is induced in the leaf of the Japanese pear when challenged by *A. alternata.* Plant defence responses are first activated in the organs located at the site of infection but are then extended to the uninfected systemic (distal) organs, activating a systemic acquired resistance (SAR) which is effective against a broad spectrum of pathogens in the whole plant [10-12]. However, limited studies have examined the induction of SAR in chrysanthemum/black spot host-pathogen interaction, so far. In the present study, the systemic responses are expected by surveying gene expression profiles in the noninfected (systemic) leaves.

RNA-Seq technology has been developed to enable the simultaneous sequencing of very large numbers of short reads, and in so doing has revolutionized the qualitative and quantitative analysis of the transcriptome [13-16]. When applied to cotton infected with a wilt pathogen, of the >32,000 genes identified by mapping the reads to a genomic sequence assembly, over 3,000 were found to be up- or down-regulated as part of the defence response [16]. Similarly, an analysis of the banana-*Fusarium oxysporum* interaction successfully demonstrated the up-regulation of genes involved in hormone synthesis, pathogenesis-related genes, transcription factors and signalling/regulatory genes [17]. Finally, the lettuce-*Botrytis cinerea* interaction has been shown to feature the induction of genes involved in the phenylpropanoid pathway and in terpenoid synthesis, as well as a global down-regulation of genes responsible for photosynthesis [18].

In present study, we aimed to 1) elucidate the localized responses to the infections on the inoculated site by comparing libraries generated from mock-inoculated and inoculated leaves; 2) to describe the systemic response by comparing libraries generated from neighbouring leaves from mock-inoculated and inoculated leaves. The present study reports the outcome of a RNA-Seq based analysis of the chrysanthemum*-A. tenuissima* interaction. The experiment has yielded information regarding the identity of the genes which are either up- or down-regulated as part of the defence response. The majority of the differentially transcribed (DT) genes were involved in either pathogen recognition, reactive oxygen species detoxification, cell wall modification or phytohormone signalling, but also a range of transcription factors, belonging to various families were identified. Validation of the RNA-Seq data was provided by subjecting a set of 23 of the DT genes to quantitative RT-PCR (qPCR).

## Results

### Analysis of RNA-Seq libraries

**Figure 1:**
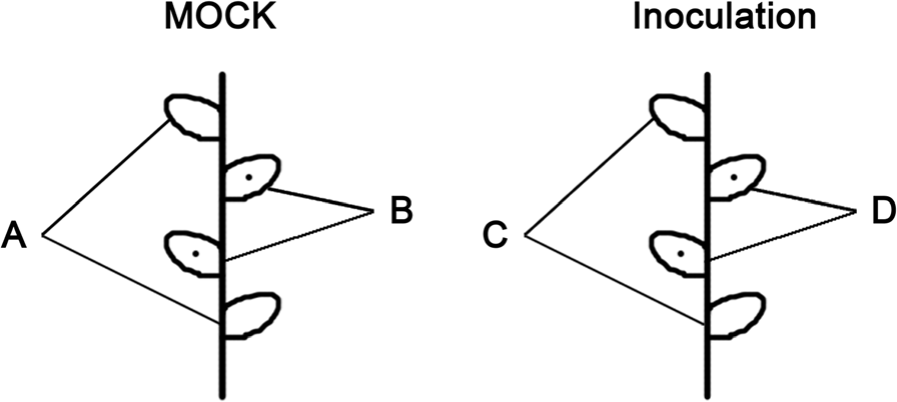
**The source of RNA libraries prepared from mock- and pathogen-inoculated chrysanthemum plants. A** and **B**: mock inoculation, **C** and **D**: inoculation with an *A. tenuissima* spore suspension. **A**, **C**: The first and fourth true leaves without any treatment. **B**: the second and third mock-inoculated true leaves. **D**: the second and third pathogen-inoculated true leaves. Leaves were harvested at 0 h, 6 h, 24 h, 48 h and 72 h after inoculation.

The major characteristics of the four libraries (Figure 1) are summarized in Table 1 and Additional file 1: Figure S1. The number of raw reads per library ranged from ~7.2 to ~7.6 million, and the total number of base pairs sequenced from 352,864,582 to 370,177,360 (Table 1) (Accession No. for library A SRS464569; Accession No. for library B SRS480632; Accession No. for library C SRS480633; Accession No. for library D SRS480635). After removal of reads including adaptor sequence, reads in which >10% of the bases were uncertain, a total of, respectively 7,524,234, 7,248,778, 7,201,318 and 7,554,640 clean reads were obtained, corresponding to 368,687,466, 355,190,122, 352,864,582 and 370,177,360 base pairs (Table 1). The proportion of clean reads was >99.30% in each library (Additional file 1: Figure S1).

**Table 1:** Summary of read mapping

**Sample ID**	**Total reads**	**Total basepairs**	**Total mapped reads**	**Perfect match**	**<=2 bp mismatch**	**Unique match**	**Multi-position match**	**Total unmapped reads**
A	7524234	368687466	5429990	3412870	2017120	3522413	1907577	2094244
	(100.00%)	(100.00%)	(72.17%)	(45.36%)	(26.81%)	(46.81%)	(25.35%)	(27.83%)
B	7248778	355190122	5069400	3184769	1884631	3321526	1747874	2179378
	(100.00%)	(100.00%)	(69.93%)	(43.94%)	(26.00%)	(45.82%)	(24.11%)	(30.07%)
C	7201318	352864582	5033405	3149786	1883619	3322965	1710440	2167913
	(100.00%)	(100.00%)	(69.90%)	(43.74%)	(26.16%)	(46.14%)	(23.75%)	(30.10%)
D	7554640	370177360	5060651	3142621	1918030	3366700	1693951	2493989
	(100.00%)	(100.00%)	(66.99%)	(41.60%)	(25.39%)	(44.56%)	(22.42%)	(33.01%)

### Read mapping

**Figure 2:**
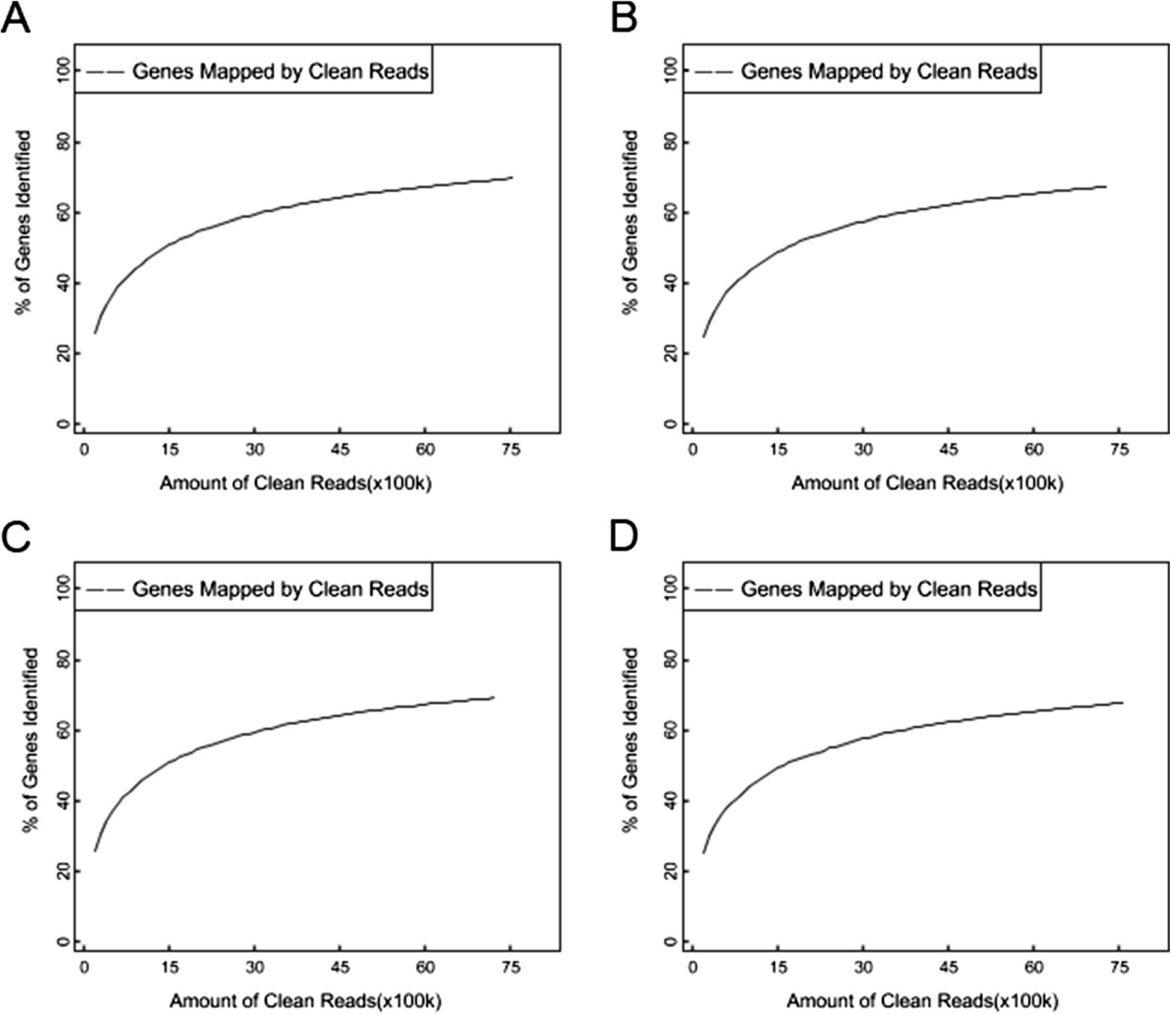
**Sequencing saturation analysis of each library.** Sequencing saturation in the four libraries of **A**, **B**, **C** and **D**. The number of different genes detected rose as the read number was increased.

A reference gene database [Raw sequence data were deposited in the NCBI Sequence Read Archive database (http://trace.ncbi.nlm.nih.gov/Traces/sra_sub/sub.cgi?) under accession number SRP029991] which included all known *Chrysanthemum morifolium* unigene sequences was used to map the RNA-Seq reads. Based on the chosen criteria, 69.75% of the clean reads recognized sequences in this database (Table 1). On a per library basis, the proportions of the clean reads uniquely mapped to the database were, respectively, 46.81%, 45.82%, 46.14% and 44.56%. In addition, the proportion of the clean reads from library D uniquely mapped to the publicly available *A.tenuissima* database was 0.02%. The number of genes identified increased with the number of reads, but above 6,000,000 reads no further genes were detected, implying full saturation of the transcriptome (Figure 2).

### GO classification of DT genes

**Figure 3:**
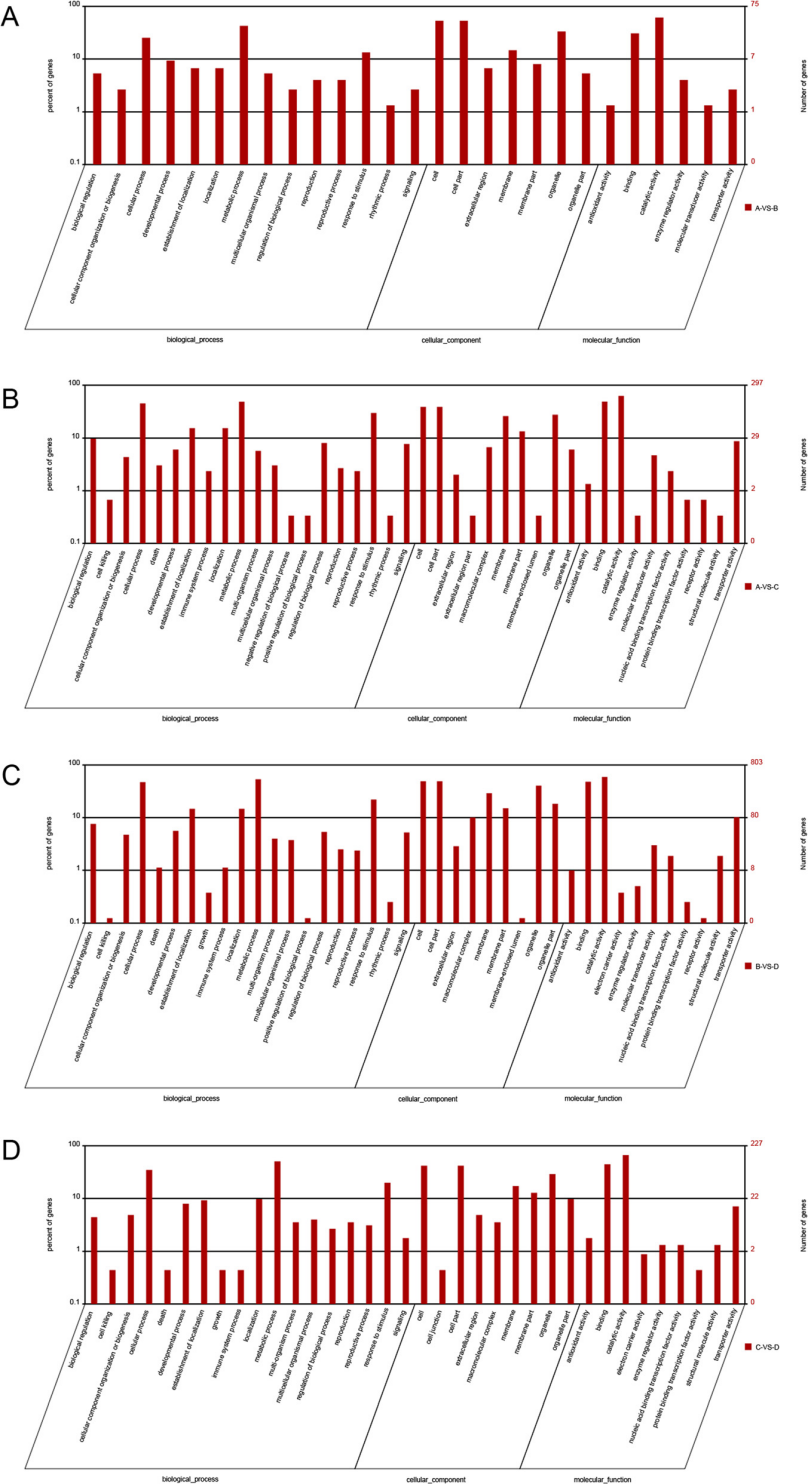
**Gene Ontology (GO) classifications of DT genes.** DT genes were annotated in three categories: biological process, cellular component and molecular function. Y-axis (right) represents the number of DT genes in each category; Y-axis (left) represents the percentage of a specific category of DT genes within that main category. Panels **A**, **B**, **C** and **D** (left) represents DT genes in the contrast between library A (leaf 1 and 4 of plants mock-inoculated on leaf 2 and 3) and B (mock-treated leaf 2 and 3) (A-VS-B) (right) , library A and C (leaf 1 and 4 of plants inoculated by the pathogen on leaf 2 and 3) (A-VS-C) (right), library B and D (pathogen-infected leaf 2 and 3) (B-VS-D) (right), library C and D (C-VS-D) (right), respectively.

Of the 217 genes classified as DT genes in the contrast between library A (leaf 1 and 4 of plants mock-inoculated on leaf 2 and 3) and B (mock-treated leaf 2 and 3), 106 could be assigned a GO classification; the equivalent number for the A *vs* C (leaf 1 and 4 of plants inoculated by the pathogen on leaf 2 and 3) contrast was 418 out of 659, for the B *vs* D (pathogen-infected leaf 2 and 3) contrast 1,057 out of 1,705, and for the C *vs* D contrast 294 out of 494 (Additional file 2: Table S1, Additional file 3: Table S2, Additional file 4: Table S3 and Additional file 5: Table S4, Additional file 6: Table S5, Additional file 7: Table S6, Additional file 8: Table S7 and Additional file 9: Table S8). Furthermore, of the 659 genes classified as DT genes in the contrast between library A and C (A *vs* C), 469 (71.2%) behaved similarly between A *vs* C and B *vs* D; the equivalent number for the B *vs* D contrast was 469 out of 1705 (27.5%) (Additional file 7: Table S6 and Additional file 8: Table S7). For the A *vs B* contrast, seven genes were categorized as “cellular component”, six as “molecular function” and 14 as “biological process”; the respective distributions in A *vs* C, B *vs* D and C *vs* D were ten, ten and 20, nine, 11 and 20, and nine, nine and 18 (Figure 3). The frequency of DT genes was highest in the contrast B *vs* D. The commonest molecular functions of the DT genes in this contrast were binding and catalytic activity; in terms of cellular component, most were associated with cells, cell parts, macromolecular complexes, membranes, membrane parts, organelles and organelle parts; finally, in terms of biological process, the majority were associated with cellular processes, metabolic processes and the response to stimulus (Figure 3). Signalling responses to or mediated by JA, SA and ET were well represented, particularly those active in JA-mediated signalling. Some transcription factors and cell wall modification genes were also differentially transcribed, as were genes involved in secondary metabolism (phenylpropanoid pathway and terpenoid synthesis). Among the biological processes well represented among the down-regulated genes were those involved in photosynthesis. In the contrast between B and D, a small number of up-regulated genes belonged to the categories ‘cell killing’, ‘positive regulation of biological process’, ‘membrane-enclosed lumen’ and ‘receptor activity’. A higher number of genes identified in this contrast were associated with ‘response to stimulus’ and ‘signalling’ than in the contrasts A *vs* B, A *vs* C and C *vs* D (Figure 3).

### Changes in transcription level

**Figure 4:**
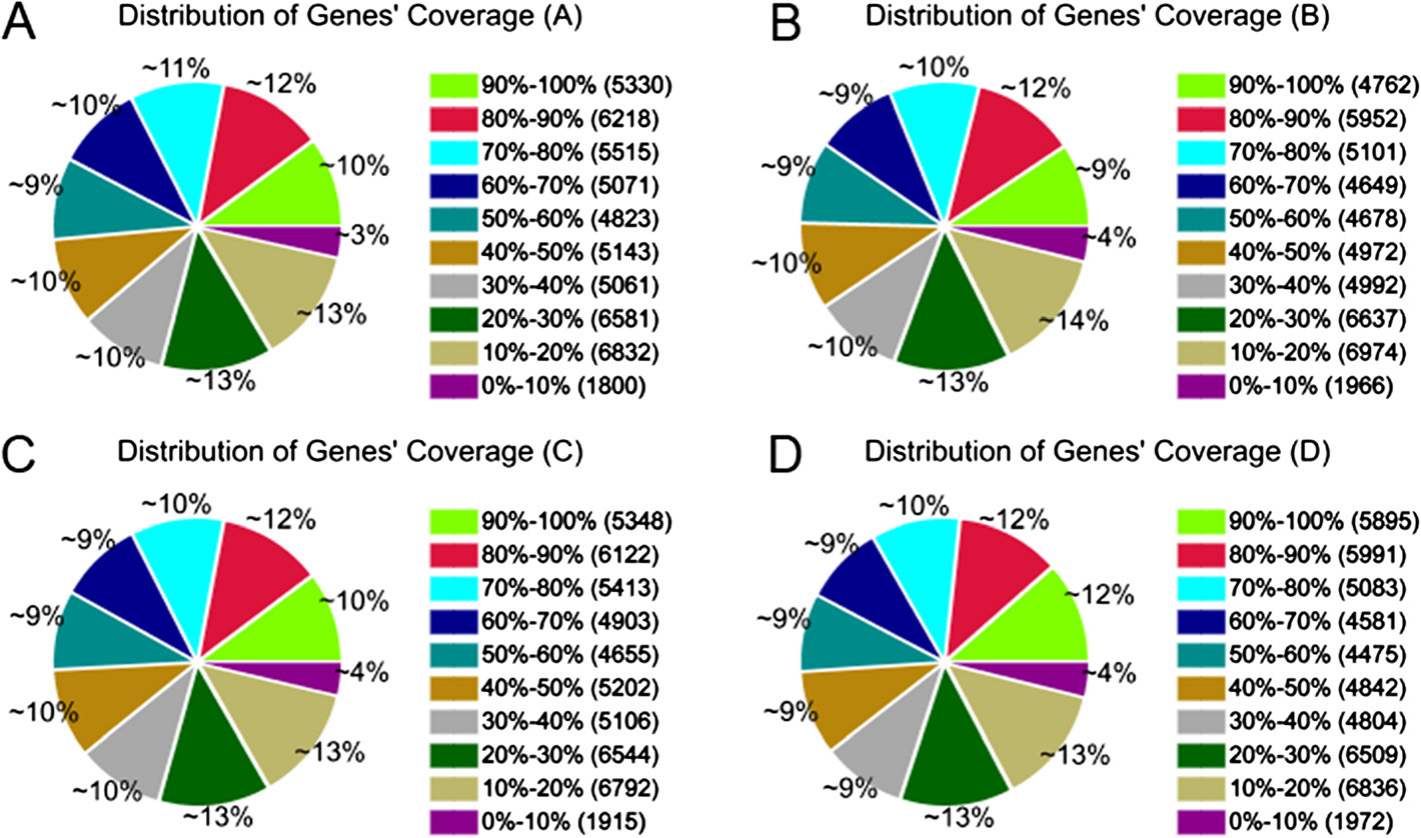
**Distribution of gene coverage analysis of each library.** Distribution of gene coverage in libraries **A**, **B**, **C** and **D**.

**Figure 5:**
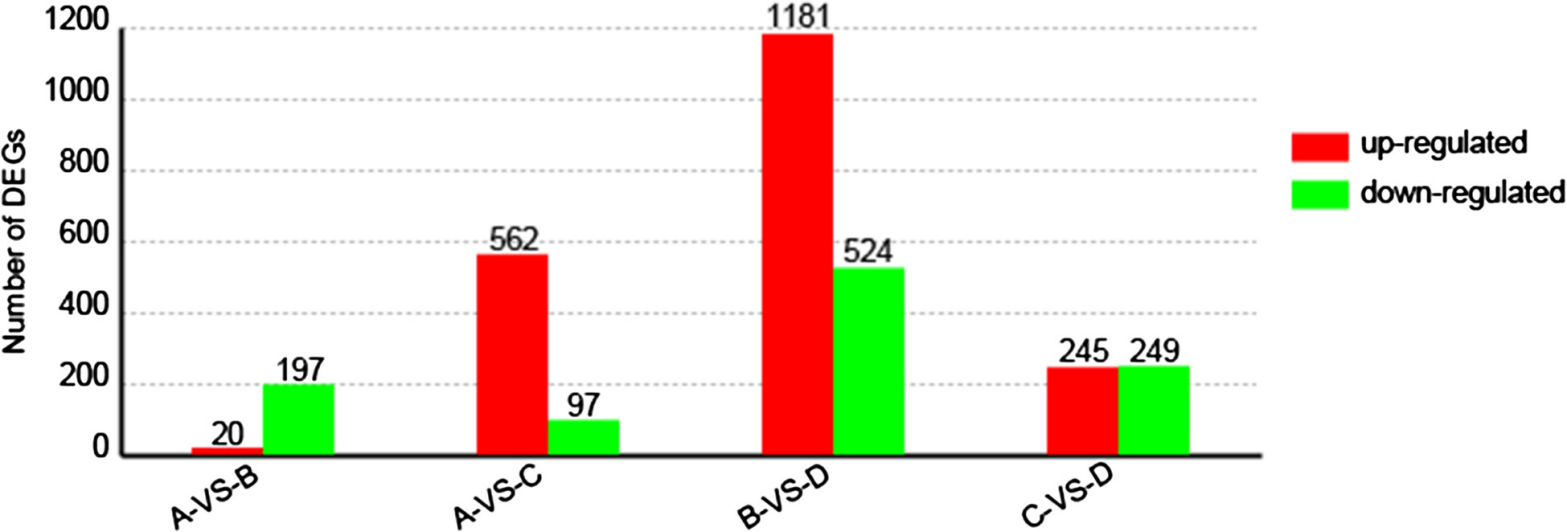
The number of DT unigenes identified in each library contrast.

The distribution of unigene coverage in each sample was analysed as a way of evaluating the quality of the RNA-Seq dataset (Figure 4). The term “gene coverage” reflects the proportion of the full gene sequence represented by RNA-Seq reads. For most of the unigenes, gene coverage was >50%. The transcription level of each unigene (Additional file 10: Table S9) was derived from the number of relevant reads recovered following Mortazavi *et al.* (2008) [19]. DT genes (Additional file 6: Table S5, Additional file 7: Table S6, Additional file 8: Table S7, Additional file 9: Table S8) were identified using an algorithm developed by Audic *et al.* (1997) [20]. Between A and B, 20 genes were up- and 197 down-regulated, between A and C, the totals were 562 and 97, respectively, between B and D, 1,181 and 524, respectively and C and D, 245 and 249, respectively (Figure 5).

### Transcription factors, cell wall modification genes and genes involved in JA and SA signalling were all regulated by *A. tenuissima* inoculation

The transcription data indicated that infection by *A. tenuissima* regulated a number of transcription factors and genes associated with pathogenesis and JA and SA signalling. Three members of the GRAS-type transcription factor family were more abundantly transcribed in the inoculated than in the mock-inoculated leaves. When qPCR was performed on a selection of the 23 of the DT genes to validate the conclusions drawn from the RNA-Seq analysis, all the genes behaved as predicted. Most of the genes examined (the exception was the *MYB* transcription factor CL9570) were induced after *A. tenuissima* inoculation (Table 2). The qPCR analysis suggested that genes involved in cell wall modification, JA and SA signalling and transcription factors comprised a network of interactions, providing the host with a capacity to fine-tune its disease response.

**Table 2:** **Genes differentially transcribed in ‘Zaoyihong’ leaves in response to mock-inoculation (B) and *A. tenuissima *** **inoculation (D)**

**GeneID**	**Function**	**Annotation**	**B-**	**D-**	**B-Relative**	**D-Relative**
			**RPKM**	**RPKM**	**level of gene expression**	**level of gene expression**
	Transcription factors					
Unigene6575_All		MYB family transcription factor	16.44	78.47	38.91 ± 4.27	128.30 ± 8.75
Unigene16113_All		MYB family transcription factor	4.16	15.43	2.12 ± 0.25	5.53 ± 0.11
CL9570		MYB family transcription factor	40.79	16.56	20.65 ± 0.61	9.22 ± 0.34
Unigene29332_All		Ethylene-responsive transcription factor	19.13	95.07	52.19 ± 2.11	134.46 ± 9.55
Unigene17395_All		Ethylene-responsive transcription factor	8.06	29.67	1.93 ± 0.16	8.52 ± 0.21
Unigene271_All		Ethylene-responsive transcription factor	29.14	105.39	18.36 ± 1.63	59.55 ± 2.61
Unigene7360_All		WRKY family transcription factor	140.72	1061.25	91.14 ± 4.36	766.75 ± 123.16
Unigene12209_All		WRKY family transcription factor	45.59	291.26	35.82 ± 2.34	253.35 ± 8.63
Unigene37863_All		WRKY family transcription factor	100.02	506.41	56.99 ± 3.64	305.58 ± 11.64
Unigene37669_All		WRKY family transcription factor	251.80	974.58	159.19 ± 15.47	722.79 ± 37.85
Unigene23051_All		NAC transcription factor	18.01	101.86	14.91 ± 1.26	78.02 ± 3.25
Unigene4479_All		NAC transcription factor	14.71	43.49	14.59 ± 1.37	39.91 ± 1.47
CL14412		NAC transcription factor	33.29	99.47	1.81 ± 0.11	22.31 ± 2.11
Unigene41060_All		GRAS family transcription factor	17.43	94.16	49.72 ± 5.56	166.22 ± 8.29
Unigene20543_All		GRAS family transcription factor	72.90	271.62	52.64 ± 4.53	169.66 ± 7.79
Unigene11471_All		GRAS family transcription factor	15.68	38.84	63.39 ± 4.99	285.75 ± 22.55
	JA signaling pathway					
Unigene11800_All		JAZ gene	20.57	97.20	56.24 ± 2.76	254.24 ± 17.19
Unigene3689_All		MYC2 transcription factor	71.44	27.57	34.38 ± 2.78	14.16 ± 0.74
CL10952		Vegetative storage protein	65.82	133.03	114.24 ± 4.80	224.06 ± 16.05
	SA signaling pathway					
Unigene23699_All		NPR1 gene	20.05	45.55	147.74 ± 18.02	187.23 ± 7.05
Unigene52251_All		TGA transcription factor	7.64	49.89	3.88 ± 0.44	21.00 ± 0.79
	Cell wall modification					
CL11209		Polygalacturonase-inhibiting protein	1.03	30.96	1.01 ± 0.07	15.04 ± 1.08
Unigene9160_All		Polyphenol oxidase	87.35	485.99	99.99 ± 26.96	499.20 ± 24.85

All these genes were significantly up- or down-regulated (*P* value < 0.05). The RPKM data were obtained from the RNA-Seq analysis and the relative level of transcript abundance from the qPCR analysis.

## Discussion

### Global patterns of transcription in response to infection by *A. tenuissima*

The chrysanthemum genome is polyploid and large, so has not as yet benefited from comprehensive and integrated genomic and transcriptomic sequence analysis. The molecular basis of its defence response against pathogen infection is currently poorly understood, but the advent of high-throughput sequencing technology now allows an unprecedented opportunity to explore it. About 70% of the reads in each of the four RNA-Seq libraries were mappable back to known transcripts (Table 1), a proportion which is somewhat lower than achieved in the lettuce/*B. cinerea* system [18], probably reflecting the more comprehensive status of the lettuce transcriptome. The 30% of reads which were not mappable are presumably associated with as yet unidentified transcripts [16]. The validation through qPCR of the transcripts identified as regulated by *A. tenuissima* infection showed that the RNA-Seq method is well suited for the analysis of transcription induced as part of the defence response in chrysanthemum (Table 2). In all, 659 DT genes were identified in the A *vs* C contrast and 1,705 in B *vs* D (Figure 5, Tables S7, S8). A comparison with the outcomes of the lettuce/*B. cinerea* and *A. thaliana/B. cinerea* interactions [18,21,22] showed that only two genes were up-regulated in all three systems, namely *Lsa004290*.1, *At1g74360* and *Unigene7965_All* (encoding an LRR protein kinase) and *Lsa016859.1*, *At4g17500* and *Unigene17395_All* (ERF1). During the early phase of both the lettuce/*B. cinerea* and the *A. thaliana/B. cinerea* interactions, genes in the ET pathway are heavily involved in the defence response, a finding which was replicated in the chrysanthemum/*A. tenuissima* interaction. The RNA-Seq data further show that a large number of genes are involved in the host response between six and 72 h post inoculation, including several genes involved in the JA and SA pathways. Some of these are discussed in more detail below.

### Pathogen recognition-related genes modulated by *A. tenuissima* infection

Plant pattern recognition receptors (PRRs) perceive microbe-associated molecular patterns (MAMPs), a set of molecular signatures encompassing whole classes of microbes. This recognition initiates a basal level of immunity (termed MAMP-triggered immunity) [23]. Receptor-like kinases (RLKs), which form the largest plant receptor family, are PRRs localized at the plasma membrane [24]. The involvement of RLKs in certain host-pathogen interactions has been well documented experimentally [18]. The RNA-Seq-based transcriptomic analysis of the lettuce/*B. cinerea* interaction has revealed that several types of RLK are differentially transcribed [18], and the same phenomenon was recorded in the chrysanthemum/*A. tenuissima* interaction. The damage caused by microbes can induce the host to synthesize MAMP-like products, termed damage-associated molecular patterns (DAMPs) [23]. Oligogalacturonides (OGs) released from the plant cell wall activate the DAMP-associated response. The induction of an OG receptor wall-associated kinase (WAK) has been suggested as being necessary for the survival *A. thaliana* challenged by pathogen infection [25]. Five WAK-like kinases (encoded by *Unigene52017_All*, *Unigene36001_All*, *Unigene55763_All*, *Unigene49198_All* and *Unigene12436_All*) were among the DT genes detected in the B *vs* D contrast (Additional file 11: Table S10), and a sixth (*Unigene49198_All*) was identified in the A *vs* C contrast (Additional file 12: Table S11). Another study showed that WAKs were involved in the immune responses against *B. cinerea*. Furthermore, transgenic plants overexpressing WAK1 conferred resistance to *B. cinerea* in *A. thaliana*[26].

Genes encoding a second class of PRR, the leucine-rich repeat RLKs (*LRR-RLK*s), are known to be involved in both basal and cultivar-specific host immunity [27]. In *A. thaliana*, *BRI1* encodes an LRR-RLK which forms a heterodimeric complex with a second LRR-RLK called BAK1 acting as a negative regulator of microbial infection-induced cell death [27]. *Bak1* mutants develop spreading necrosis after the triggering of apoptosis by infection with *B. cinerea*[27]. The transcription of two *LRR-RLK* (*Unigene20925_All* and *Unigene27322_All*), four *BRI-like* (*Unigene55939_All*, *Unigene15368_All*, *Unigene29292_All*, *Unigene18133_All*), six *BAK1* genes (*Unigene37501_All*, *Unigene36228_All*, *Unigene16709_All*, *Unigene16958_All*, *Unigene14705_All*, and *Unigene27008_All*), and two somatic embryogenesis receptor kinase (*SERK*) genes (*Unigene22508_All*, *Unigene14416_All*) was modulated by *A. tenuissima* infection in the B *vs* D contrast (Additional file 13: Table S12), as were an additional two *BRI-like* (*Unigene15368_All* and *Unigene4877_All*), a somatic embryogenesis receptor kinase (*SERK*) genes (Unigene22508_All), and four *BAK1* genes (*Unigene36228_All*, *Unigene4877_All*, *Unigene14705_All* and *Unigene27008_All*) in the A *vs* C contrast (Additional file 12: Table S11). All the above genes were up-regulated except for *Unigene18133_All*. In the lettuce/*B. cinerea* interaction, one *BRI-like* (*Lsa034184.1*) gene was up-regulated, but no *BAK1* genes [18].

The products of genes encoding cysteine-rich receptor-like kinases (*CRKs*) are RLKs which contain an extracellular cysteine-rich repeat domain. These genes are reportedly activated by oxidative stress, pathogen attack and exposure to SA [18,28-30]. Although less widely researched, they have been characterized to be involved in the pathogen defense and programmed cell death in *A. thaliana*[28,30]. Here, three *CRKs w*ere identified as DT genes (*Unigene55939_All*, *Unigene15489_All* and *Unigene14705_All*) in the B *vs* D comparison (Additional file 14: Table S13) and a further three (*Unigene4299_All*, *Unigene14705_All* and *Unigene15489_All*) in the A *vs* C comparison (Additional file 15: Table S14). Thus, *PRR-RLKs* are clearly involved in the chrysanthemum host response to *A. tenuissima* infection. The early activation of these genes may reflect their transcription as an attempt by the host to recognize MAMPs/DAMPs.

### ROS (reactive oxygen species) detoxification genes modulated by *A. tenuissima* infection

MAMPs are known to trigger the production of ROS in response to pathogen infection, largely derived from NADPH oxidase activity (commonly referred to as “respiratory burst oxidase homologues” (rboh)) [17,31]. Two chrysanthemum rboh homologues were recognized: *Unigene300_All* (homologue of *rbohD*) and *Unigene45792_All* (*rbohF*); both were differentially transcribed in the B *vs* D (Additional file 16: Table S15) and A *vs* C contrasts (Additional file 17: Table S16). The lettuce *rbohD* homologue (*Lsa002796.1*) was induced 48 h after infection with *B. cinerea*, but its *rbohF* homologue *(Lsa018309.1*) was not up-regulated [18]. In *A. thaliana*, both *rbohD* and *rbohF* are required for ROS detoxification [32]. Two further pathogen-inducible α-dioxygenases (*Unigene32071_All* and *Unigene12359_All*) (Additional file 16: Table S15) were differentially transcribed in the B *vs* D contrast, and one (*Unigene32071_All*) in A *vs* C (Additional file 17: Table S16); this class of gene was also up-regulated in the lettuce/*B. cinerea* system; its product is involved in protecting the cell against oxidative stress [18].

### Genes associated with photosynthesis were mostly down-regulated by *A. tenuissima* infection

DT genes involved in photosynthesis were uniformly down-regulated in the B *vs* D contrast, with the sole exception of *Unigene2020_All* (Additional file 18: Table S17). In the contrast C *vs* D, four photosynthesis-related DT genes were detected, and all were down-regulated (Additional file 19: Table S18). The response mirrors the outcomes in the lettuce/*B. cinerea* and lettuce/*Verticillium dahliae* systems [18,33], as well as in other documented plant-pathogen interactions [34-39]. *Unigene6198*_*All*, predicted to be involved in the determination of the plant’s circadian clock, was down-regulated in both the B *vs* D (Additional file 18: Table S17) and C *vs* D contrasts (Additional file 19: Table S18). Similar examples of the suppression of such genes by pathogen infection have been described in both lettuce [18] and *A. thaliana*[40]. In the case of *A. thaliana*, *B. cinerea* infection appears to dampen the oscillating transcription of certain core clock components, leading to the suggestion that the pathogen attempts to dampen the host’s defence response, because a set of genes associated with plant immune responses was revealed to be regulated by the plant’s circadian clock [41].

### Genes associated with cell wall protection affected by *A. tenuissima* infection

Both *PGIP* and *PPO* were up-regulated in response to *A. tenuissima* infection (Table 2). *PGIP* and *PPO* are known to respond to various cues [42-48], including the presence of *A. solani*[42], *A. triticina*[49], *A. macrospora*[50], *Sclerospora graminicola*[51] and *Colletotrichum lindemuthianum*[52]. *PGIP* transcript abundance increased over the period 6–24 h after inoculation with *A. tenuissima* (Table 2). Many fungi secrete endo-polygalacturonase, an enzyme which degrades the polysaccharides present in the plant cell wall. Host genes involved in the early defence response include those which help to reinforce the cell wall, and thereby inhibit pathogen entry. PGIP’s role in mediating resistance to *A. alternata* infection has been shown in both rough lemon and Japanese pear [9,53], while the growth of *B. cinerea* is restricted in transgenic tomato heterologously expressing pear *PGIP*[54]. *PPO* transcription in chrysanthemum was enhanced after inoculation (Table 2), similar to what has been observed in the leaf of Japanese pear inoculated with *A. alternata*[9]. In tomato, the constitutive expression of *PPO* increases host resistance to *Pseudomonas syringae*, while its down-regulation enhances susceptibility [55,56].

### JA and SA signalling pathway-related genes involved in the response to *A. tenuissima* infection

*CmJAZ* (*Unigene11800*_*All*), *CmMYC2* (*Unigene3689*_*All*), *CmVSP* (*CL10952*), *CmNPR1* (*Unigene23699_All*) and *CmTGA* (*Unigene52251_All*) were all induced by *A. tenuissima* infection (Table 2). NPR1 is a major component of SA signalling, functioning as a co-activator of the TGA transcription factors known to regulate the transcription of various SA-responsive genes [57]. JAZ (jasmonate ZIM domain) proteins repress JA signalling by binding to transcriptional regulators such as MYC2. The degradation of JAZ proteins relieves the JAZ-mediated repression of the JA signalling pathway and thereby activates a large number of JA responsive genes [57,58]. JA and SA both play an important part in host defence against herbivorous insects and microbial pathogens. In *A. thaliana*, VSP2 and PR-1 are, respectively, JA- and SA-responsive proteins [58]. Overall, a number of genes within the JA and SA signalling pathways are involved in the defence response of chrysanthemum against *A. tenuissima* infection.

### Transcription factors responding to *A. tenuissima* infection

Transcription factors are central to the control of the timing and placement of defence response gene expression [59]. Their mode of action is to first recognize and then bind to regulatory elements located in the promoter region of their target genes, thereby activating or de-activating their transcription. Here, five classes of transcription factor (MYB, AP2/ERF, WRKY, NAC and GRAS) were identified among the DT genes responding to *A. tenuissima* infection. In *A. thaliana*, *AtMYB30*, *AtMYB44* and *AtMYB96* are all involved in the triggering of apoptosis and therefore resistance against biotrophic bacterial pathogens such as *Pseudomonas syringae*[60-64]. *AtMYB108* is required for resistance against *B. cinerea* and *A. brassicicola*[65]. The transcription of *AtMYB58* has been associated with secondary cell wall formation [66]. Here, one *CmMYB* copy (*Unigene6575_All* and a homologue of *AtMYB58*), was prominently transcribed from 48 h after inoculation with *A. tenuissima* (Table 2). Therefore, it was speculated that CmMYB (Unigene6575_All) may be involved in defence response to the necrotrophic fungus *A. tenuissima* through the regulation of secondary cell wall biosynthesis. Modifications to the plant cell wall were already recognized as the potential mechanism of resistance, which was previously provided by the reports on the response of plants to fungal challenges [67]. A second *CmMYB* (*Unigene16113_All*, homologous to *AtMYB74*) was also up-regulated by *A. tenuissima* infection (Table 2). In *A. thaliana*, *AtMYB74* reportedly responds to salinity stress and the exogenous supply of abscisic acid, ETH and JA [68], but not as yet to pathogen infection. Therefore, it was speculated that this gene may be involved in defense response to the necrotrophic fungus *A. tenuissima* and may be also associated with ABA and JA signaling in Chrysanthemum. Other transcription factors in the AP2/ERF, WRKY, NAC and GRAS families were abundantly transcribed in the leaf following *A. tenuissima* inoculation. Many reports have also indicated that four families of transcription factors: MYB proteins, ethylene-responsive-element-binding factors (ERF), WRKY proteins and NAC proteins link to plant stress responses, such as pathogens [59,60,69-72]. Little evidence has been provided to date regarding the participation of GRAS transcription factors in the defence response.

In conclusion, it was clear that infection with *A. tenuissima* induced a wide range of genes in the chrysanthemum leaf. The response involved a complex set of interactions between pathogenesis-related genes, genes in the JA and SA signalling pathway and transcription factors. A more detailed understanding of the identity of these genes will help to unravel the molecular basis of the defence response of chrysanthemum to *A. tenuissima* infection, and eventually lead to the recognition of candidates for the targeted genetic improvement of chrysanthemum.

## Conclusions

In this study, we characterized the leaf transcriptome of chrysanthemum and provided the comparative DT genes involeved in the interaction between chrysanthemum and *A. tenuissima*. These findings provide a substantial contribution to existing sequence resources of chrysanthemum, and a strong basis for further characterization of gene expression profiles in the interaction of chrysanthemum and *A. tenuissima*. The majority of the DT genes were those involved in pathogen recognition, reactive oxygen species detoxification, cell wall modification, phytohormone signalling, and transcription factors belonging to various families were also identified, which will improve our understanding of the molecular mechanisms underlining direct response and induced systemic resistance of chrysanthemum to *A. tenuissim*.

## Methods

### Plant materials, *A. tenuissima* inoculum preparation and inoculation

The chrysanthemum variety ‘Zaoyihong’ was obtained from the Chrysanthemum Germplasm Resource Preserving Centre, Nanjing Agricultural University, China. Uniform cuttings were propagated in sand, and rooted seedlings transplanted into a 2:1 mixture of garden soil and vermiculite without fertilizer supplementation. The plants were grown under a 16 h photoperiod with a day/night temperature of, respectively, 25°C and 18°C. The relative humidity was maintained at 68-75% [3]. *A. tenuissima* conidia were isolated from diseased chrysanthemum plants, and cultured on potato dextrose agar at 25°C. An aqueous suspension of 10^6^ spores per ml was prepared with a few drops of Triton X-100 added as a wetting agent [3]. The surface of the second and third true leaves of 20 day old root cuttings was punctured with a needle (approximately 0.30 mm diameter), and a 10 μl droplet of spore suspension was placed on the puncture site. Mock treatments comprised 10 μl droplets of sterile distilled water. After inoculation, the plants were held at 100% relative humidity and 25°C in the dark for 24 h, and then illuminated with 120 μmol m^-2^ s^-1^ cool white fluorescent light with a 12 h photoperiod. The leaves of three seedlings were sampled for each treatment at 0 h, 6 h, 24 h, 48 h and 72 h after inoculation. The samples collected at defined time points of each treatment were pooled for RNA-seq.

### RNA extraction

Four separate libraries (A-D, see Figure 1) were prepared. Extracts of the second and the third true leaves of mock-treated and pathogen-infected plants gave rise to, respectively, libraries B and D, while extracts of the first and the fourth noninfected (systemic) true leaves of plants gave rise to libraries A and C. Total RNA was isolated using a Total RNA Isolation System (Takara, Japan), following the manufacturer’s recommendations. The quality of the total RNA (RNA Integrity Number > 6.5 and 28S:18S > 1.0) was verified using a 2100 Bioanalyzer RNA Nano chip device (Agilent, Santa Clara, CA, USA) and its concentration ascertained using an ND-1000 spectrophotometer (NanoDrop, Wilmington, DE). The standards applied were 1.8 ≤ OD_260/280_ ≤ 2.2 and OD_260/230_ ≥ 1.8. At least 10 μg RNA was pooled in an equimolar fashion from each of the three sampled plants [73].

### cDNA library construction and Illumina sequencing

Each total RNA extract was first treated with RNase-free DNase I (TaKaRa, Dalian, China) to remove contaminating DNA, and the mRNA content was concentrated by capturing on magnetic oligo (dT) beads. The mRNA was fragmented to a size of ~200 bp using a fragmentation buffer, and the resulting fragments used to synthesize the first cDNA strand by priming with random hexamers. The second strand was generated using a SuperScript Double-Stranded cDNA Synthesis kit (Invitrogen, Camarillo, CA), purified via magnetic beads, the ends repaired and a single adenine base added to the 3′ ends. Sequencing adaptors were then ligated to the fragments, and agarose gel electrophoresis used to select the range of fragments suitable for PCR amplification. Sequencing using an Illumina HiSeq™ 2000 platform was performed at the Beijing Genomics Institute (Shenzhen, China; http://www.genomics.cn/index.php), following the manufacturer’s protocols.

### Treatment of sequence data

Raw reads were saved as .fastq files, and filtered to remove adaptor sequences, reads in which the proportion of non-called bases was > 10% and reads in which low quality (≤ 5) bases represented > 50% of the read. The remaining reads were mapped onto the set of chrysanthemum unigene sequences using SOAPaligner/SOAP2 [74]. A maximum of two mismatches was permitted for the purpose of alignment. The frequency of occurrence of individual reads was normalized to RPKM (reads per kb per million reads) [19]. Differential transcription between pathogen-inoculated and mock samples was based on the log_2_ ratio of the two RPKM values. All raw RNA-Seq data have been deposited at the sequence read archive (SRA) of NCBI (Additional file 20: Table S19).

### Identification of DT genes

Following Audic and Claverie (1997) [20], a stringent algorithm was developed to identify DT genes. The FDR (false discovery rate) provides a criterion to determine the *P*-value threshold in multiple tests and analyses by manipulating the FDR value. Here, differential transcription was declared provided that the *P*-value was < 0.05, the FDR ≤ 0.001 and the absolute value of log_2_ induction ratios of treated samples compared with mock-treatment or control (CK) was ≥ 1.0. Standard gene ontology (GO) was used to describe DT gene functionality, and a hypergeometric test was used to map the DT genes to GO terms based on the BGI WEGO (Web Gene Ontology Annotation Plot, http://wego.genomics.org.cn/cgi-bin/wego/index.pl).

### Quantitative real-time PCR (qPCR) validation

The transcription of 23 selected candidate genes was determined using quantitative real time PCR (qPCR). The samples collected at different time points were pooled, which were used for qPCR analysis, contaminating DNA removed by RNase-free DNase I treatment and the first cDNA strand synthesized using a Super RT kit (BioTeke, Beijing, China). A set of gene-specific primer pairs (sequences given in Table 3) was designed using Primer3 software [18,75]. qPCRs were based on SYBR_Green I (TOYOBO’, Japan) implemented in a Rotor-Gene 3000 device (Corbett, Australia). The chrysanthemum *EF1α* gene was used as a reference. Each 25 μl qPCR reaction contained 10 μl SYBR Green PCR master mix, 0.2 μM of each primer and 10 ng cDNA, and the amplification regime consisted of an initial denaturation of 95°C/60 s, followed by 40 cycles of 95°C/15 s, 55°C/15 s, 72°C/20 s. Transcript abundances are given as the mean ± SE of three replicates. Relative transcription levels were calculated using the 2^-△△CT^ method [76].

**Table 3:** Primers of quantitative reverse transcription-polymerase chain reaction for validation of the digital gene expression data

**Gene ID**	**Primer F (5′-3′)**	**Primer R (5′-3′)**	**Basic annotation**
Unigene6575_All	CGGAATCAAGAAAGGTGCAT	ATTCATCCATCGTAGCCTGC	MYB family transcription factor
Unigene16113_All	CGTTGTGACGAGAGGAGTGA	CTTCTTCGGATGGGAATTGA	MYB family transcription factor
CL9570	AAAATGCTGGATTGCTGAGG	AAGACCATTTGTTGCCAAGG	MYB family transcription factor
Unigene29332_All	CAATTTCATTACCGAGGCGT	TGCCTAGCCATCTTCGAGTT	Ethylene-responsive transcription factor
Unigene17395_All	ACGAGATCCCAACAAAGGTG	TTGGACCCTTTGAAACGAAG	Ethylene-responsive transcription factor
Unigene271_All	CTCTAACTCCGTGCCTCGTC	TTAGAGGGGTTAGGAGGCGT	Ethylene-responsive transcription factor
Unigene7360_All	CACCTCGTGGTAGTGGGAGT	TGACTGCCATTATCGGTCAA	WRKY family transcription factor
Unigene12209_All	GTGGCTGAGATTGGTGGTTT	GCCTTTACAAGCGTTTCAGC	WRKY family transcription factor
Unigene37863_All	GGATCATCTCAGGGGGATTT	GACAACAGCATCACCACCAC	WRKY family transcription factor
Unigene37669_All	TAGATGCACATACCGCAAGG	TTTTCAGGCAAAGGTGGTTC	WRKY family transcription factor
Unigene23051_All	ACCTACAAACATCGGCTTCG	CTTCGAAAATGGTGCCCTAA	NAC transcription factor
Unigene4479_All	CAGCCAAAAATACCCTGCAT	ACGGACGAAAAACTGATTGG	NAC transcription factor
CL14412	AGTGTCCCTTCACCACCTTCG	CCGTTTCTCGTGACCCTGTT	NAC transcription factor
Unigene41060_All	GTAATCTGGAGCATGGGTGG	CTTAATGGTGTGCCCGTTTC	GRAS family transcription factor
Unigene20543_All	CTTGTTGCGAAACTGAGCTG	GACCCCTGCTGAAAAGATGA	GRAS family transcription factor
Unigene11471_All	TCGAGGGGAAGGAAACACTA	GGTTCTTCACAAGCCGACAT	GRAS family transcription factor
Unigene11800_All	AAATGTTTCGGTTTTCGACG	GATCTTTGTTAGGTGCCGGA	JAZ gene
Unigene3689_All	CATTGATGTAACAAACGGCG	AACCAACGCTTCTACACGCT	MYC2 transcription factor
CL10952	CCATTGACCCAAGCAACTTT	TTGAATACGGCTCCTCCATC	Vegetative storage protein
Unigene23699_All	CAAAGCATTGGATTGTGACG	TCTAAGATTGACATCCGCCC	NPR1 gene
Unigene52251_All	CCGGCTTTGATTGAACTGAT	CGTGGTTTCGTGGGTAGAGT	TGA transcription factor
CL11209	GCCGTATAATCGCTCTCACC	CCGGTGAGGTTTGTCAGTTT	Polygalacturonase-inhibiting protein
Unigene9160_All	TGAACACAACCAAGATGGGA	CCTCCACCTTGTCCACTGTT	Polyphenol oxidase

## Competing interests

The authors declare that they have no competing interests.

## Authors’ contributions

LHY and SAP contributed to RACE PCR, real-time PCR, bioinformatics analysis and writing of the manuscript. CFD, JJF and CSM conceived of the study, and participated in its design and contributed to revisions of the manuscript. FWM and GZY participated in experiment materials preparation. WHB helped with the RNA extraction. All authors read and approved the final manuscript.

## Supplementary Material

Additional file 1: Figure S1 Composition of the raw reads in the four RNA libraries. “Clean” reads are those remaining after removal of adaptor sequences, reads in which the proportion of non-called bases was >10% and reads in which low quality (≤ 5) bases represented >50% of the reads. The numbers in parentheses indicate the percentage of each type of read present. Click here for file

Additional file 2: Table S1 GO classification of the genes differentially transcribed in the contrast between libraries A and B. Click here for file

Additional file 3: Table S2 GO classification of the genes differentially transcribed in the contrast between libraries A and C. Click here for file

Additional file 4: Table S3 GO classification of the genes differentially transcribed in the contrast between libraries B and D. Click here for file

Additional file 5: Table S4 GO classification of the genes differentially transcribed in the contrast between libraries C and D. Click here for file

Additional file 6: Table S5 Genes differentially transcribed in the contrast between libraries A and B. The criteria applied for assigning significance were: *P-*value < 0.05, FDR ≤ 0.001, and estimated absolute |log_2_Ratio(B/A)| ≥ 1. Genes listed in descending order of absolute |log_2_Ratio(B/A)|. GeneIDs retrieved from the Chrysanthemum Reference Sequence Database. Annotation of unigene sequences performed using BlastX (E <10). The “GeneLength” column gives the length of exon sequence. A- and B expression: frequency of unigene transcripts in libraries A and B, respectively. A- and B-RPKM: reads per kb per million reads for each unigene in libraries A and B, respectively. Log_2_ Ratio(B/A): the ratio between the RPKM in B and the RPKM in A. Up-Down-Regulation(B/A), *P*-value and FDR of each gene are also shown. KEGG: annotation according to the KEGG database by BLAST. Blast nr: identification of homologues in GenBank. GO Component, GO Function and Go Process: ontology information of Cellular Components, Molecular Function and Biological Processes of Gene-corresponding GO terms. “-”: no hit. Click here for file

Additional file 7: Table S6 Genes differentially transcribed in the contrast between libraries A and C. The criteria applied for assigning significance were: *P-*value < 0.05, FDR ≤ 0.001, and estimated absolute |log_2_Ratio(C/A)| ≥ 1. Genes listed in descending order of absolute |log_2_Ratio(C/A)|. GeneIDs retrieved from the Chrysanthemum Reference Sequence Database. Annotation of unigene sequences performed using BlastX (E <10). The “GeneLength” column gives the length of exon sequence. A- and C expression: frequency of unigene transcripts in libraries A and C, respectively. A- and C–RPKM: reads per kb per million reads for each unigene in libraries A and C, respectively. Log_2_ Ratio(C/A): the ratio between the RPKM in C and the RPKM in A. Up-Down-Regulation(C/A), *P*-value and FDR of each gene are also shown. KEGG: annotation according to the KEGG database by BLAST. Blast nr: identification of homologues in GenBank. GO Component, GO Function and Go Process: ontology information of Cellular Components, Molecular Function and Biological Processes of Gene-corresponding GO terms. “-”: no hit. Click here for file

Additional file 8: Table S7 Genes differentially transcribed in the contrast between libraries B and D. The criteria applied for assigning significance were: *P-*value < 0.05, FDR ≤ 0.001, and estimated absolute |log_2_Ratio(D/B)| ≥ 1. Genes listed in descending order of absolute |log_2_Ratio(D/B)|. GeneIDs retrieved from the Chrysanthemum Reference Sequence Database. Annotation of unigene sequences performed using BlastX (E <10). The “GeneLength” column gives the length of exon sequence. B- and D expression: frequency of unigene transcripts in libraries B and D, respectively. B- and D-RPKM: reads per kb per million reads for each unigene in libraries B and D, respectively. Log_2_ Ratio(D/B): the ratio between the RPKM in D and the RPKM in B. Up-Down-Regulation(D/B), *P*-value and FDR of each gene are also shown. KEGG: annotation according to the KEGG database by BLAST. Blast nr: identification of homologues in GenBank. GO Component, GO Function and Go Process: ontology information of Cellular Components, Molecular Function and Biological Processes of Gene-corresponding GO terms. “-”: no hit. Click here for file

Additional file 9: Table S8 Genes differentially transcribed in the contrast between libraries C and D. The criteria applied for assigning significance were: *P-*value < 0.05, FDR ≤ 0.001, and estimated absolute |log_2_Ratio(D/C)| ≥ 1. Genes listed in descending order of absolute |log_2_Ratio(D/C)|. GeneIDs retrieved from the Chrysanthemum Reference Sequence Database. Annotation of unigene sequences performed using BlastX (E <10). The “GeneLength” column gives the length of exon sequence. C– and D expression: frequency of unigene transcripts in libraries C and D, respectively. C- and D-RPKM: reads per kb per million reads for each unigene in libraries C and D, respectively. Log_2_ Ratio(D/C): the ratio between the RPKM in D and the RPKM in C. Up-Down-Regulation(D/C), *P*-value and FDR of each gene are also shown. KEGG: annotation according to the KEGG database by BLAST. Blast nr: identification of homologues in GenBank. GO Component, GO Function and Go Process: ontology information of Cellular Components, Molecular Function and Biological Processes of Gene-corresponding GO terms. “-”: no hit. Click here for file

Additional file 10: Table S9 The transcription level of each unigene derived from the number of relevant reads recovered in the four libraries. The “GeneLength” column gives the length of exon sequence. Click here for file

Additional file 11: Table S10 The differential transcription of Wall-associated receptor kinase-like (*WAK-like*) genes in the contrast B *vs* D. The criteria applied for assigning significance were: *P*-value < 0.05, FDR ≤ 0.001, and estimated absolute |log_2_^Ratio(D/B)^| ≥ 1. RPKM: reads per kb per million reads. Click here for file

Additional file 12: Table S11 The differential transcription of Wall-associated receptor kinase-like (*WAK-like*), brassinosteroid insensitive 1 (*BRI-like*), somatic embryogenesis receptor kinase (*SERK*), and BRI1-associated receptor kinase 1 (*BAK1*) genes in the contrast A *vs* C. The criteria applied for assigning significance were: *P*-value < 0.05, FDR ≤ 0.001, and estimated absolute |log_2_^Ratio(C/A)^| ≥ 1. RPKM: reads per kb per million reads. Click here for file

Additional file 13: Table S12 The differential transcription of leucine-rich repeat receptor-like kinase (*LRR-RLK*), brassinosteroid insensitive 1 (*BRI-like*), BRI1-associated receptor kinase 1 (*BAK1*), and somatic embryogenesis receptor kinase (*SERK*) genes in the contrast B *vs* D. The criteria applied for assigning significance were: *P*-value < 0.05, FDR ≤ 0.001, and estimated absolute |log_2_^Ratio(D/B)^| ≥ 1. RPKM: reads per kb per million reads. Click here for file

Additional file 14: Table S13 The differential transcription of cysteine-rich receptor-like protein kinase (*CRKs*) genes in the contrast B *vs* D. The criteria applied for assigning significance were: *P*-value < 0.05, FDR ≤ 0.001, and estimated absolute |log_2_^Ratio(D/B)^| ≥ 1. RPKM: reads per kb per million reads. Click here for file

Additional file 15: Table S14 The differential transcription of cysteine-rich receptor-like protein kinase (*CRKs*) genes in the contrast A *vs* C. The criteria applied for assigning significance were: *P*-value < 0.05, FDR ≤ 0.001, and estimated absolute |log_2_^Ratio(C/A)^| ≥ 1. RPKM: reads per kb per million reads. Click here for file

Additional file 16: Table S15 The differential transcription of respiratory burst oxidase, and alpha-dioxygenase genes in the contrast B *vs* D. The criteria applied for assigning significance were: *P*-value < 0.05, FDR ≤ 0.001, and estimated absolute |log_2_^Ratio(D/B)^| ≥ 1. RPKM: reads per kb per million reads. Click here for file

Additional file 17: Table S16 The differential transcription of respiratory burst oxidase, and alpha-dioxygenase genes in the contrast A *vs* C. The criteria applied for assigning significance were: *P*-value < 0.05, FDR ≤ 0.001, and estimated absolute |log_2_^Ratio(C/A)^| ≥ 1. RPKM: reads per kb per million reads. Click here for file

Additional file 18: Table S17 The differential transcription of photosynthesis and circadian rhythm-related genes in the contrast B *vs* D. The criteria applied for assigning significance were: *P*-value < 0.05, FDR ≤ 0.001, and estimated absolute |log_2_^Ratio(D/B)^| ≥ 1. RPKM: reads per kb per million reads. Click here for file

Additional file 19: Table S18 The differential transcription of photosynthesis and circadian rhythm-related genes in the contrast C *vs* D. The criteria applied for assigning significance were: *P*-value < 0.05, FDR ≤ 0.001, and estimated absolute |log_2_^Ratio(D/C)^| ≥ 1. RPKM: reads per kb per million reads. Click here for file

Additional file 20: Table S19 High-throughput sequencing metadata. Click here for file
