# Supplementary figures and images for: RNA-Seq derived identification of differential transcription in the chrysanthemum leaf following inoculation with Alternaria tenuissima
Source: BMC Genomics. 2014 Jan 4;15:9. doi: 10.1186/1471-2164-15-9 (PMC3890596; doi:10.1186/1471-2164-15-9)

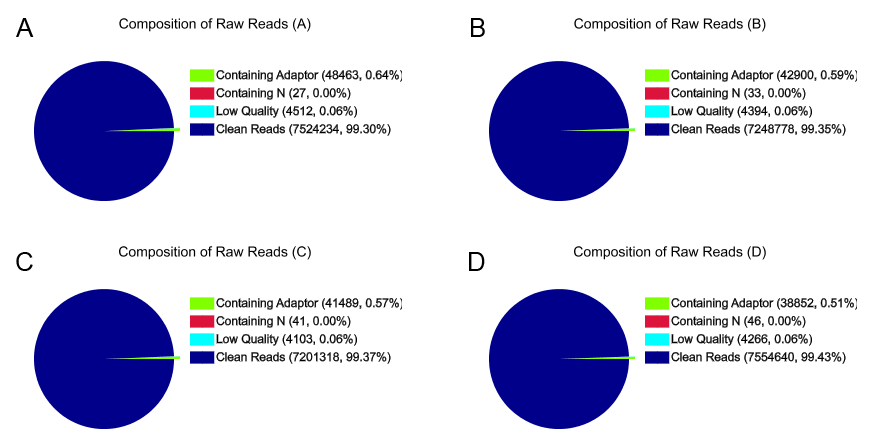

Supplement: Additional file 1: Figure S1 — Composition of the raw reads in the four RNA libraries. “Clean” reads are those remaining after removal of adaptor sequences, reads in which the proportion of non-called bases was >10% and reads in which low quality (≤ 5) bases represented >50% of the reads. The numbers in parentheses indicate the percentage of each type of read present. [file 1471-2164-15-9-S1.tiff]
