# Supplementary material for: RNA-Seq derived identification of differential transcription in the chrysanthemum leaf following inoculation with Alternaria tenuissima
Source: BMC Genomics. 2014 Jan 4;15:9. doi: 10.1186/1471-2164-15-9 (PMC3890596; doi:10.1186/1471-2164-15-9)
Supplement: Additional file 11: Table S10 — The differential transcription of Wall-associated receptor kinase-like (WAK-like) genes in the contrast B vs D. The criteria applied for assigning significance were: P-value < 0.05, FDR ≤ 0.001, and estimated absolute |log2Ratio(D/B)| ≥ 1. RPKM: reads per kb per million reads. [file 1471-2164-15-9-S11.doc]

Additional file 11: Table S10 The differential transcription of Wall-associated receptor kinase-like (*WAK-like*) genes in the contrast B *vs* D. The criteria applied for assigning significance were: *P*-value < 0.05, FDR ≤ 0.001, and estimated absolute |log_2_^Ratio(D/B)^| ≥ 1. RPKM: reads per kb per million reads.

| GeneID | B-RPKM | D-RPKM | log_2_ Ratio(D/B) | Up-Down-  Regulation(D/B) | *P*-value | FDR | Gene description |
| --- | --- | --- | --- | --- | --- | --- | --- |
| Unigene52017_All | 15.04 | 56.10 | 1.90 | Up | 9.57E-12 | 3.52E-10 | WAK-like kinase |
| Unigene36001_All | 9.92 | 36.52 | 1.88 | Up | 1.23E-05 | 0.000224 | wall-associated receptor kinase-like 20 isoform 2 |
| Unigene55763_All | 14.82 | 55.40 | 1.90 | Up | 4.78E-08 | 1.23E-06 | wall-associated receptor kinase 2-like |
| Unigene49198_All | 29.94 | 69.51 | 1.22 | Up | 3.95E-07 | 9.08E-06 | wall-associated receptor kinase 8-like |
| Unigene12436_All | 25.52 | 55.47 | 1.12 | Up | 1.39E-08 | 3.77E-07 | wall-associated kinase |
