# Supplementary material for: RNA-Seq derived identification of differential transcription in the chrysanthemum leaf following inoculation with Alternaria tenuissima
Source: BMC Genomics. 2014 Jan 4;15:9. doi: 10.1186/1471-2164-15-9 (PMC3890596; doi:10.1186/1471-2164-15-9)
Supplement: Additional file 12: Table S11 — The differential transcription of Wall-associated receptor kinase-like (WAK-like), brassinosteroid insensitive 1 (BRI-like), somatic embryogenesis receptor kinase (SERK), and BRI1-associated receptor kinase 1 (BAK1) genes in the contrast A vs C. The criteria applied for assigning significance were: P-value < 0.05, FDR ≤ 0.001, and estimated absolute |log2Ratio(C/A)| ≥ 1. RPKM: reads per kb per million reads. [file 1471-2164-15-9-S12.doc]

Additional file 12: Table S11. The differential transcription of Wall-associated receptor kinase-like (*WAK-like*), brassinosteroid insensitive 1 (*BRI-like*), somatic embryogenesis receptor kinase (*SERK*), and BRI1-associated receptor kinase 1 (*BAK1*) genes in the contrast A *vs* C. The criteria applied for assigning significance were: *P*-value < 0.05, FDR ≤ 0.001, and estimated absolute |log_2_^Ratio(C/A)^| ≥ 1. RPKM: reads per kb per million reads.

| GeneID | A-RPKM | C-RPKM | log_2_ Ratio(C/A) | Up-Down-  Regulation(C/A) | *P*-value | FDR | Gene description |
| --- | --- | --- | --- | --- | --- | --- | --- |
| Unigene49198_All | 28.61 | 59.84 | 1.06 | Up | 1.66E-05 | 0.000548 | wall-associated receptor kinase 8-like |
| Unigene15368_All | 5.96 | 16.97 | 1.51 | Up | 1.56E-08 | 8.55E-07 | protein brassinosteroid insensitive 1 |
| Unigene4877_All | 101.38 | 243.83 | 1.27 | Up | 3.61E-18 | 4.12E-16 | protein brassinosteroid insensitive 1 |
| Unigene22508_All | 5.66 | 35.76 | 2.66 | Up | 9.13E-08 | 4.51E-06 | somatic embryogenesis receptor kinase 1 |
| Unigene36228_All | 13.00 | 78.19 | 2.59 | Up | 1.99E-29 | 3.61E-27 | brassinosteroid insensitive 1-associated receptor kinase 1 |
| Unigene4877_All | 101.38 | 243.83 | 1.27 | Up | 3.61E-18 | 4.12E-16 | brassinosteroid insensitive 1-associated receptor kinase 1 precursor |
| Unigene14705_All | 12.52 | 30.03 | 1.26 | Up | 6.11E-06 | 0.000223 | brassinosteroid insensitive 1-associated receptor kinase 1 |
| Unigene27008_All | 7.53 | 17.29 | 1.20 | Up | 9.56E-06 | 0.000331 | brassinosteroid insensitive 1-associated receptor kinase 1 |
