# Supplementary material for: RNA-Seq derived identification of differential transcription in the chrysanthemum leaf following inoculation with Alternaria tenuissima
Source: BMC Genomics. 2014 Jan 4;15:9. doi: 10.1186/1471-2164-15-9 (PMC3890596; doi:10.1186/1471-2164-15-9)
Supplement: Additional file 13: Table S12 — The differential transcription of leucine-rich repeat receptor-like kinase (LRR-RLK), brassinosteroid insensitive 1 (BRI-like), BRI1-associated receptor kinase 1 (BAK1), and somatic embryogenesis receptor kinase (SERK) genes in the contrast B vs D. The criteria applied for assigning significance were: P-value < 0.05, FDR ≤ 0.001, and estimated absolute |log2Ratio(D/B)| ≥ 1. RPKM: reads per kb per million reads. [file 1471-2164-15-9-S13.doc]

Additional file 13: Table S12. The differential transcription of leucine-rich repeat receptor-like kinase (*LRR-RLK*), brassinosteroid insensitive 1 (*BRI-like*), BRI1-associated receptor kinase 1 (*BAK1*), and somatic embryogenesis receptor kinase (*SERK*) genes in the contrast B *vs* D. The criteria applied for assigning significance were: *P*-value < 0.05, FDR ≤ 0.001, and estimated absolute |log_2_^Ratio(D/B)^| ≥ 1. RPKM: reads per kb per million reads.

| GeneID | B-RPKM | D-RPKM | log_2_ Ratio(D/B) | Up-Down-  Regulation(D/B) | *P*-value | FDR | Gene description |
| --- | --- | --- | --- | --- | --- | --- | --- |
| Unigene20925_All | 8.25 | 22.87 | 1.47 | Up | 2.34E-05 | 0.000405 | leucine-rich repeat receptor-like kinase |
| Unigene27322_All | 23.91 | 52.01 | 1.12 | Up | 7.70E-14 | 3.38E-12 | probably leucine-rich repeat receptor-like protein |
| Unigene55939_All | 12.71 | 46.48 | 1.87 | Up | 2.61E-06 | 5.38E-05 | protein brassinosteroid insensitive 1 |
| Unigene15368_All | 5.13 | 15.63 | 1.61 | Up | 2.50E-08 | 6.58E-07 | protein brassinosteroid insensitive 1 |
| Unigene29292_All | 12.95 | 28.04 | 1.11 | Up | 2.45E-06 | 5.06E-05 | protein brassinosteroid insensitive 1 |
| Unigene18133_All | 19.13 | 5.11 | -1.90 | Down | 9.73E-11 | 3.31E-09 | protein brassinosteroid insensitive 1 |
| Unigene37501_All | 2.80 | 19.66 | 2.81 | Up | 1.04E-05 | 0.000192 | brassinosteroid insensitive 1-associated receptor kinase 1 |
| Unigene36228_All | 20.61 | 125.87 | 2.61 | Up | 4.10E-45 | 5.80E-43 | brassinosteroid insensitive 1-associated receptor kinase 1 |
| Unigene16709_All | 2.27 | 11.39 | 2.33 | Up | 3.92E-05 | 0.000647 | brassinosteroid insensitive 1-associated receptor kinase 1 precursor, putative |
| Unigene16958_All | 35.59 | 103.67 | 1.54 | Up | 5.03E-34 | 5.26E-32 | brassinosteroid insensitive 1-associated receptor kinase 1 |
| Unigene14705_All | 14.49 | 36.19 | 1.32 | Up | 4.31E-07 | 9.85E-06 | brassinosteroid insensitive 1-associated receptor kinase 1 |
| Unigene27008_All | 7.22 | 16.15 | 1.16 | Up | 4.21E-05 | 0.00069 | brassinosteroid insensitive 1-associated receptor kinase 1 |
| Unigene22508_All | 14.68 | 72.83 | 2.31 | Up | 4.09E-12 | 1.56E-10 | somatic embryogenesis receptor kinase 1 |
| Unigene14416_All | 21.18 | 49.61 | 1.23 | Up | 1.57E-06 | 3.32E-05 | somatic embryogenesis receptor kinase 4 |
