# Supplementary material for: RNA-Seq derived identification of differential transcription in the chrysanthemum leaf following inoculation with Alternaria tenuissima
Source: BMC Genomics. 2014 Jan 4;15:9. doi: 10.1186/1471-2164-15-9 (PMC3890596; doi:10.1186/1471-2164-15-9)
Supplement: Additional file 14: Table S13 — The differential transcription of cysteine-rich receptor-like protein kinase (CRKs) genes in the contrast B vs D. The criteria applied for assigning significance were: P-value < 0.05, FDR ≤ 0.001, and estimated absolute |log2Ratio(D/B)| ≥ 1. RPKM: reads per kb per million reads. [file 1471-2164-15-9-S14.doc]

Additional file 14: Table S13. The differential transcription of cysteine-rich receptor-like protein kinase (*CRKs*) genes in the contrast B *vs* D. The criteria applied for assigning significance were: *P*-value < 0.05, FDR ≤ 0.001, and estimated absolute |log_2_^Ratio(D/B)^| ≥ 1. RPKM: reads per kb per million reads.

| GeneID | B-RPKM | D-RPKM | log_2_ Ratio(D/B) | Up-Down-  Regulation(D/B) | *P*-value | FDR | Gene description |
| --- | --- | --- | --- | --- | --- | --- | --- |
| Unigene55939_All | 12.71 | 46.48 | 1.87 | Up | 2.61E-06 | 5.38E-05 | cysteine-rich receptor-like protein kinase 10-like |
| Unigene15489_All | 16.80 | 60.82 | 1.86 | Up | 6.41E-33 | 6.42E-31 | cysteine-rich receptor-like protein kinase 42 |
| Unigene14705_All | 14.49 | 36.19 | 1.32 | Up | 4.31E-07 | 9.85E-06 | cysteine-rich receptor-like protein kinase 10-like |
