# Supplementary material for: RNA-Seq derived identification of differential transcription in the chrysanthemum leaf following inoculation with Alternaria tenuissima
Source: BMC Genomics. 2014 Jan 4;15:9. doi: 10.1186/1471-2164-15-9 (PMC3890596; doi:10.1186/1471-2164-15-9)
Supplement: Additional file 15: Table S14 — The differential transcription of cysteine-rich receptor-like protein kinase (CRKs) genes in the contrast A vs C. The criteria applied for assigning significance were: P-value < 0.05, FDR ≤ 0.001, and estimated absolute |log2Ratio(C/A)| ≥ 1. RPKM: reads per kb per million reads. [file 1471-2164-15-9-S15.doc]

Additional file 15: Table S14. The differential transcription of cysteine-rich receptor-like protein kinase (*CRKs*) genes in the contrast A *vs* C. The criteria applied for assigning significance were: *P*-value < 0.05, FDR ≤ 0.001, and estimated absolute |log_2_^Ratio(C/A)^| ≥ 1. RPKM: reads per kb per million reads.

| GeneID | A-RPKM | C-RPKM | log_2_ Ratio(C/A) | Up-Down-  Regulation(C/A) | *P*-value | FDR | Gene description |
| --- | --- | --- | --- | --- | --- | --- | --- |
| Unigene4299_All | 25.74 | 82.57 | 1.68 | Up | 4.57E-11 | 3.22E-09 | cysteine-rich receptor-like protein kinase 10-like |
| Unigene14705_All | 12.52 | 30.03 | 1.26 | Up | 6.11E-06 | 0.000223 | cysteine-rich receptor-like protein kinase 10-like |
| Unigene15489_All | 17.63 | 39.17 | 1.15 | Up | 8.33E-12 | 6.32E-10 | cysteine-rich receptor-like protein kinase 42 |
