# Supplementary material for: RNA-Seq derived identification of differential transcription in the chrysanthemum leaf following inoculation with Alternaria tenuissima
Source: BMC Genomics. 2014 Jan 4;15:9. doi: 10.1186/1471-2164-15-9 (PMC3890596; doi:10.1186/1471-2164-15-9)
Supplement: Additional file 16: Table S15 — The differential transcription of respiratory burst oxidase, and alpha-dioxygenase genes in the contrast B vs D. The criteria applied for assigning significance were: P-value < 0.05, FDR ≤ 0.001, and estimated absolute |log2Ratio(D/B)| ≥ 1. RPKM: reads per kb per million reads. [file 1471-2164-15-9-S16.doc]

Additional file 16: Table S15. The differential transcription of respiratory burst oxidase, and alpha-dioxygenase genes in the contrast B *vs* D. The criteria applied for assigning significance were: *P*-value < 0.05, FDR ≤ 0.001, and estimated absolute |log_2_^Ratio(D/B)^| ≥ 1. RPKM: reads per kb per million reads.

| GeneID | B-RPKM | D-RPKM | log_2_ Ratio(D/B) | Up-Down-  Regulation(D/B) | *P*-value | FDR | Gene description |
| --- | --- | --- | --- | --- | --- | --- | --- |
| Unigene300_All | 12.45 | 72.29 | 2.54 | Up | 3.23E-39 | 3.95E-37 | Respiratory burst oxidase homolog protein D |
| Unigene45792_All | 9.10 | 39.50 | 2.12 | Up | 2.83E-15 | 1.36E-13 | Respiratory burst oxidase homolog protein F |
| Unigene32071_All | 28.93 | 116.87 | 2.01 | Up | 5.39E-20 | 3.29E-18 | pathogen-inducible alpha-dioxygenase |
| Unigene12359_All | 46.82 | 169.12 | 1.85 | Up | 6.35E-91 | 1.84E-88 | alpha-dioxygenase |
