# Supplementary material for: RNA-Seq derived identification of differential transcription in the chrysanthemum leaf following inoculation with Alternaria tenuissima
Source: BMC Genomics. 2014 Jan 4;15:9. doi: 10.1186/1471-2164-15-9 (PMC3890596; doi:10.1186/1471-2164-15-9)
Supplement: Additional file 17: Table S16 — The differential transcription of respiratory burst oxidase, and alpha-dioxygenase genes in the contrast A vs C. The criteria applied for assigning significance were: P-value < 0.05, FDR ≤ 0.001, and estimated absolute |log2Ratio(C/A)| ≥ 1. RPKM: reads per kb per million reads. [file 1471-2164-15-9-S17.doc]

Additional file 17: Table S16. The differential transcription of respiratory burst oxidase, and alpha-dioxygenase genes in the contrast A *vs* C. The criteria applied for assigning significance were: *P*-value < 0.05, FDR ≤ 0.001, and estimated absolute |log_2_^Ratio(C/A)^| ≥ 1. RPKM: reads per kb per million reads.

| GeneID | A-RPKM | C-RPKM | log_2_ Ratio(C/A) | Up-Down-  Regulation(C/A) | *P*-value | FDR | Gene description |
| --- | --- | --- | --- | --- | --- | --- | --- |
| Unigene300_All | 12.92 | 47.02 | 1.86 | Up | 8.19E-19 | 9.61E-17 | Respiratory burst oxidase homolog protein D |
| Unigene45792_All | 5.48 | 24.96 | 2.19 | Up | 7.05E-11 | 4.91E-09 | Respiratory burst oxidase homolog protein F |
| Unigene32071_All | 25.83 | 61.17 | 1.24 | Up | 2.07E-06 | 8.21E-05 | pathogen-inducible alpha-dioxygenase |
