# Supplementary material for: RNA-Seq derived identification of differential transcription in the chrysanthemum leaf following inoculation with Alternaria tenuissima
Source: BMC Genomics. 2014 Jan 4;15:9. doi: 10.1186/1471-2164-15-9 (PMC3890596; doi:10.1186/1471-2164-15-9)
Supplement: Additional file 18: Table S17 — The differential transcription of photosynthesis and circadian rhythm-related genes in the contrast B vs D. The criteria applied for assigning significance were: P-value < 0.05, FDR ≤ 0.001, and estimated absolute |log2Ratio(D/B)| ≥ 1. RPKM: reads per kb per million reads. [file 1471-2164-15-9-S18.doc]

Additional file 18: Table S17. The differential transcription of photosynthesis and circadian rhythm-related genes in the contrast B *vs* D. The criteria applied for assigning significance were: *P*-value < 0.05, FDR ≤ 0.001, and estimated absolute |log_2_^Ratio(D/B)^| ≥ 1. RPKM: reads per kb per million reads.

| GeneID | B-RPKM | D-RPKM | log_2_ Ratio(D/B) | Up-Down-  Regulation(D/B) | *P*-value | FDR | Gene description |
| --- | --- | --- | --- | --- | --- | --- | --- |
| Unigene2020_All | 23.32 | 49.99 | 1.10 | Up | 8.46E-10 | 2.62E-08 | heterotrophic ferredoxin 2 |
| Unigene28981_All | 51.17 | 12.98 | -1.98 | Down | 2.97E-21 | 1.93E-19 | oxygen-evolving enhancer protein 3-2, chloroplastic |
| Unigene16922_All | 56.96 | 16.15 | -1.82 | Down | 8.33E-26 | 6.36E-24 | oxygen-evolving enhancer protein 3-1, chloroplastic |
| Unigene3209_All | 803.76 | 249.50 | -1.69 | Down | 9.89E-265 | 9.14E-262 | ferredoxin |
| Unigene12252_All | 2305.28 | 788.59 | -1.55 | Down | 0 | 0 | plastocyanin, chloroplastic isoform 1 |
| Unigene8187_All | 43.24 | 14.86 | -1.54 | Down | 2.04E-12 | 8.06E-11 | ferredoxin 1 |
| Unigene29179_All | 961.55 | 336.29 | -1.52 | Down | 0 | 0 | photosystem II 22kDa protein |
| Unigene2120_All | 453.33 | 168.99 | -1.42 | Down | 1.46E-125 | 6.00E-123 | photosystem II Psb27 protein |
| Unigene11275_All | 662.77 | 254.48 | -1.38 | Down | 2.43E-205 | 1.62E-202 | photosystem I reaction center subunit |
| Unigene7949_All | 1179.75 | 454.39 | -1.38 | Down | 0 | 0 | photosystem I psaH protein |
| Unigene6395_All | 1383.49 | 548.21 | -1.34 | Down | 0 | 0 | cytochrome b6-f complex iron-sulfur subunit, chloroplastic isoform 1 |
| Unigene1939_All | 110.34 | 44.51 | -1.31 | Down | 3.16E-27 | 2.55E-25 | photosystem II 13kDa protein |
| Unigene45117_All | 83.34 | 33.67 | -1.31 | Down | 7.04E-21 | 4.47E-19 | photosystem I subunit II |
| Unigene19985_All | 784.70 | 336.01 | -1.22 | Down | 5.81E-149 | 2.93E-146 | photosystem I reaction center V |
| Unigene5042_All | 1259.59 | 553.28 | -1.19 | Down | 8.96E-279 | 9.37E-276 | photosystem II PsbY protein |
| Unigene11331_All | 1978.51 | 869.30 | -1.19 | Down | 0 | 0 | photosystem II oxygen-evolving enhancer protein 2 |
| Unigene7947_All | 668.71 | 294.21 | -1.18 | Down | 7.22E-136 | 3.25E-133 | photosystem I subunit PsaO |
| Unigene11779_All | 608.14 | 269.02 | -1.18 | Down | 8.72E-85 | 2.39E-82 | photosystem I reaction center subunit X psaK |
| Unigene11286_All | 813.76 | 361.94 | -1.17 | Down | 2.13E-187 | 1.34E-184 | F-type H^+^-transporting ATPase subunit b |
| Unigene8291_All | 1152.65 | 526.31 | -1.13 | Down | 8.00E-307 | 9.43E-304 | photosystem I subunit III |
| Unigene28972_All | 1238.77 | 569.25 | -1.12 | Down | 7.27E-185 | 4.43E-182 | photosystem I subunit IV |
| Unigene8134_All | 1391.76 | 639.81 | -1.12 | Down | 6.88E-261 | 6.25E-258 | photosystem II PsbW protein |
| Unigene26218_All | 1844.79 | 873.67 | -1.08 | Down | 0 | 0 | photosystem II oxygen-evolving enhancer protein 3 |
| Unigene29026_All | 466.40 | 228.84 | -1.03 | Down | 9.96E-130 | 4.18E-127 | F-type H^+^-transporting ATPase subunit gamma |
| Unigene29138_All | 1887.37 | 939.88 | -1.01 | Down | 0 | 0 | photosystem I subunit XI |
| Unigene6198_All | 16.01 | 5.69 | -1.49 | Down | 6.22E-06 | 0.00012 | circadian rhythm-related protein |
