# Supplementary material for: RNA-Seq derived identification of differential transcription in the chrysanthemum leaf following inoculation with Alternaria tenuissima
Source: BMC Genomics. 2014 Jan 4;15:9. doi: 10.1186/1471-2164-15-9 (PMC3890596; doi:10.1186/1471-2164-15-9)
Supplement: Additional file 19: Table S18 — The differential transcription of photosynthesis and circadian rhythm-related genes in the contrast C vs D. The criteria applied for assigning significance were: P-value < 0.05, FDR ≤ 0.001, and estimated absolute |log2Ratio(D/C)| ≥ 1. RPKM: reads per kb per million reads. [file 1471-2164-15-9-S19.doc]

Additional file 19: Table S18. The differential transcription of photosynthesis and circadian rhythm-related genes in the contrast C *vs* D. The criteria applied for assigning significance were: *P*-value < 0.05, FDR ≤ 0.001, and estimated absolute |log_2_^Ratio(D/C)^| ≥ 1. RPKM: reads per kb per million reads.

| GeneID | C-RPKM | D-RPKM | log_2_ Ratio(D/C) | Up-Down-  Regulation(D/C) | *P*-value | FDR | Gene description |
| --- | --- | --- | --- | --- | --- | --- | --- |
| Unigene28981_All | 34.55 | 12.98 | -1.41 | Down | 1.07E-09 | 8.13E-08 | photosystem II oxygen-evolving enhancer protein 3 |
| Unigene16922_All | 40.63 | 16.15 | -1.33 | Down | 1.91E-12 | 1.95E-10 | photosystem II oxygen-evolving enhancer protein 3 |
| Unigene3209_All | 544.22 | 249.50 | -1.13 | Down | 1.45E-97 | 1.56E-94 | ferredoxin |
| Unigene2120_All | 343.12 | 168.99 | -1.02 | Down | 4.88E-57 | 2.61E-54 | photosystem II Psb27 protein |
| Unigene6198_All | 16.67 | 5.69 | -1.55 | Down | 2.20E-06 | 0.0001 | circadian rhythm-related protein |
